# Influence of silicon morphology on direct current plasma electrolytic oxidation process in AlSi10Mg alloy produced with laser powder bed fusion

**DOI:** 10.1038/s41598-022-18176-x

**Published:** 2022-08-22

**Authors:** L. Pezzato, C. Gennari, M. Franceschi, K. Brunelli

**Affiliations:** Department of Industrial Engineering, University of Padua, Via Marzolo 9, 35131 Padova, Italy

**Keywords:** Materials science, Metals and alloys

## Abstract

In this work, plasma electrolytic oxidation (PEO) process was applied on AlSi10Mg samples, produced with laser powder bed fusion (L-PBF), in the as printed condition and after different heat treatments, and, for comparison, on as-cast samples of AlSi10Mg. PEO process was performed in direct-current mode using high current densities and short time in a basic silicate electrolyte. For the first time, the effects of silicon morphology in L-PBF AlSi10Mg samples, in as printed condition and after different heat treatments, on the obtained PEO coating were investigated in terms of microstructure and corrosion properties. The microstructure of the substrate was characterized with optical and electron microscopy observations (optical microscopy OM, scanning electron microscopy SEM, and transmission electron microscopy TEM) and with X-ray diffraction (XRD). The analysis showed that heat treatments of annealing and solution treating modified the morphology and distribution of silicon in the samples obtained through L-PBF. The PEO coated samples were characterized with SEM, both on the surface and in the cross-section, and compositional analysis were performed with energy dispersive spectroscopy (EDS) analysis and elemental mapping. The coatings were also analyzed with XRD and the corrosion properties evaluated through electrochemical impedance spectroscopy (EIS) tests. Also microhardness tests were performed on the substrates and on the coatings. The microstructure of the coatings was strongly influenced by the silicon distribution. In particular, a non-uniform distribution of silicon and the presence of iron-rich intermetallic (obtained in the as-cast and solution treated samples) induced the formation of more porous and thinner coatings in comparison with the ones obtained in the as printed and annealed samples. The not-uniform silicon distribution produced a not-homogenous distribution of silicon into the coatings. The particular cellular structure of the as printed sample induced the formation of a coating with a higher amorphous fraction, in comparison with the ones produced on the other samples. The higher thickness and lower porosity of the coatings obtained on the annealed and as printed samples resulted in an increase of the corrosion resistance.

## Introduction

Al-Si alloys are widely employed in the automotive and aerospace sectors due to their good castability and their combination of high strength and low density^1^. Conventionally cast Al-Si alloys normally contain coarse, acicular silicon (eutectic silicon) and Mg-containing and Fe-containing precipitates are also generally present^2^. In the last years, the interest of different industrial sectors for additive manufacturing (AM) technologies rapidly increased, mainly due to the possibility of producing complex and customized parts without a remarkable increase in costs related to dies or tools^3^. Among the different AM technologies, Laser Powder Bed Fusion (L-PBF) is one of the most promising for the possibility of obtaining fully dense metallic structures using a large variety of metal powders^4^. In comparison with traditional casting, Al-Si alloys produced by L-PBF are characterized by a completely different microstructure, due to the higher heating and cooling rates (10^3^–10^5^ K/s)^5^. In particular, the microstructure of AlSi10Mg produced with L-PBF consist of primary α-Al matrix with cellular-dendritic microstructure and eutectic microstructure with very fine fibrous Si^6^.

However, the α-Al matrix is in super-saturated solid solution condition and when the material is subjected to high temperatures, for example when heat treatments are performed, this microstructure becomes unstable and can change significantly in comparison with the one observable in the as printed condition^7^. Among the different heat treatments, the more often employed in aluminum alloys are solution treatment (followed in some cases by an aging treatment) and annealing treatment. Several studies can be found in the literature regarding the microstructural evolution of AlSi10Mg after these treatments. Takata et al*.*^8^ found after annealing treatment at 300 °C the formation of finely distributed Si particles within the columnar α-Al phase due to the Si supersaturation in the columnar α-Al matrix of the as-fabricated sample. Li et al*.*^9^ found that during solution treatment, Si atoms precipitate from the supersaturated Al matrix to form small Si particles, and increasing the solution temperature, the size of the Si particles increases, whereas their number decreases. Also, Shakil et al.^10^ obtained similar results highlighting that modification of coarse and acicular Si phase (spheroidization), homogenization of the composition, and disintegration of dissolvable phases containing Mg or other trace elements are produced by solution heat treatment. Again, Takata et al*.*^8^ found after solution treatment the formation of a Fe-containing intermetallic phase (β-AlFeSi) with a rod-shaped morphology.

Considering the previously reported discussion, the main effects of heat treatments on the microstructure of L-PBF AlSi10Mg are the redistribution of silicon, the destruction of the cellular structure formed during rapid cooling, and, in the case of solution treatment, the formation of iron-rich precipitates.

Regardless of the manufacturing process (traditional casting or additive manufacturing) and the heat treatments performed, aluminum alloys are often subjected to specific surface treatments in order to increase the corrosion and wear properties and to extend their possible applications^11^.

In additive manufactured samples, the application of post-treatments in order to achieve favorable surface properties and the desired bulk performance is even more essential than in traditional manufactured samples. In fact, additive manufactures samples generally exhibit inadequate and poor surface quality in the as-built configuration^12^. The classification of the surface treatments is mainly based on the intrinsic characteristics of the applied technology and on the final effects of the treatment on the surface of AM part; in particular, the major groups are those based on “material removal”, “no material removal” and “coatings”^13^. The treatments based on material removal are mainly: machining^14^, polishing^15^, laser-based treatments^16^ and chemical treatments^17^, whereas the treatments based on no material removal are mainly rolling^18^, sand blasting^19^ and shot penning^20^. Among the different surface treatments of AM parts, of great importance are also: electro-spark deposition^21^, anodizing^22^ and Plasma Electrolytic Oxidation^23^.

In detail, PEO process is one of the most studied in the last years^24^. PEO process is a relatively innovative treatment that permits to improve several technological characteristics of different metals, in particular aluminum^25^, magnesium^26,27^ and titanium alloys^28^.

The increasing interest in this kind of coating can be related to several factors such as: the environmentally friendly nature of the electrolyte^29^; the improved corrosion^30^ and wear^31^ properties, in comparison with similar processes like anodizing; the possibility of functionalizing the coating with proper compounds coming directly from the electrolyte^32^; and the high flexibility allowing to coat samples with different microstructures, geometry and surface roughness^33,34^. This last characteristic is especially important in the formation of coatings on samples produced by AM, generally characterized by high surface roughness. Recently, the PEO process was investigated to coat samples of titanium^35^ and aluminum alloys produced by AM^36–39^.

Although the PEO process is characterized by high flexibility, the microstructure of the substrate can strongly influence the behavior of PEO coatings. Considering Al-Si alloys, Krishtal et al*.*^40^ found that an inhomogeneous distribution of Si in the substrate produced an inhomogeneous distribution of Si into the PEO layer; Li et al*.*^41^ found that a reduction in the size of the eutectic Si causes the formation of a thicker PEO layer; Wu et al*.*^42^ found that the coating is characterized by large micropores on iron-rich precipitates and it is thin with small micropores on eutectic Si. As previously described, samples produced by L-PBF are characterized by a unique microstructure that can be significantly modified, in particular regarding Si distribution, through common heat treatments such as solution treatment and annealing. Some works, in literature, investigated the PEO process on samples of AlSi10Mg obtained by L-PBF, but none of them correlated the microstructure of the PEO coatings with the one of the substrate after the heat treatments, and in detail with the variation in the silicon distribution at the nanoscale.

The aim of this work was to study the effect of silicon distribution in L-PBF AlSi10Mg samples after heat treatments on the microstructure and corrosion and mechanical properties of PEO coatings.

## Experimental

In this section will be presented the materials and methods employed in this work. In detail will be firstly presented the methods employed for the production and characterization of substrates with different silicon distribution (“Production, heat treatments, and characterization of L-PBF samples” section) and after will be discussed the methods employed in the production and characterization of Plasma Electrolytic Oxidation coatings on the previously produced substrates (“Production and characterization of PEO coatings” section).

### Production, heat treatments, and characterization of L-PBF samples

AlSi10Mg alloy samples of 3 × 2 × 0.2 cm, produced by selective Laser Powder Bed Fusion (L-PBF), were employed as substrate for PEO coatings. The additive manufactured samples were printed with a Renishaw AM400 (Renishaw S.p.A., Turin, Italy). The employed powders (provided by Renishaw, lot number UK3402) were obtained by gas-atomization and have an average grain size of 40 µm. The samples were printed with a laser power of 200 W, exposure time of 40 ms, point distance 80 µm, hatch space 80 µm and with a layer thickness of 30 µm. The samples were employed as substrate for PEO in the as printed condition and also after two heat treatments: an annealing treatment at 300 °C for 2 h with air cooling and a solution treatment at 530 °C for 6 h and water quenching. The treatments were performed using a Carbolite (Verder Scientific, Germany) tubular electrical furnace in an inert atmosphere (Ar). The different treatments were chosen, on the base of the work of Takata et al*.*^8^, to obtain different silicon distribution in the Al matrix and, consequently, to study the effect of these distributions on the morphology of the final PEO coatings. Also, a conventional cast sample of AlSi10Mg was used for comparison.

The microstructure of the AM samples was evaluated with a LEICA DMRE optical microscope (OM) (Leica Microsystem S.r.l., Milan, Italy), a Cambridge Stereoscan 440 scanning electron microscope (SEM) (Leica Microsystem S.r.l., Milan, Italy), equipped with a Philips PV9800 EDS, and a JEOL JEM 200CX transmission electron microscope (TEM) (JEOL Ltd., Tokyo, Japan).

For OM and SEM observations, the samples were polished with standard metallographic techniques (mounted in epoxy resin, ground until 4000 grit and polished with clothes with diamond suspension 6 and 1 µm, all material for metallographic preparation from Cloeren Technology S.r.l., Italy) and then etched with Graff-Sargent etch (84 mL water, 15.5 mL HNO_3_, 0.5 mL HF, 3 g CrO_3_, chemicals from Sigma Aldrich, USA). SEM images were collected in secondary electron mode. For TEM observation, thin foils were realized by mechanical grinding until a thickness of 50 µm, followed by mechanical punching to obtain 3 mm diameter disk specimens. The final polishing and etching were performed electrochemically using a twin-jet polisher STRUERS TENUPOL-3 (Struers, Copenaghen, Denmark), with 90% methanol and 10% Nitric Acid solution, at 14 V and − 40 °C. The cooling of the solution was performed with liquid nitrogen. TEM observations were performed with an acceleration voltage of 120 kV.

Phase identification was carried out through X-ray diffraction (XRD) with a Bruker D8 Advance (Bruker, USA) working at 40 kV and 40 mA and a Cu radiation tube (Kβ radiation was filtered by mean of nickel filter on the tube side). The investigated angular range was between 20° and 75°, steps scan of 0.02°, and counting time of 3 s. The obtained patterns were analyzed using High Score Plus software in order to identify the constituent phases. Also, line profile analysis was performed on the XRD patterns in order to evaluate Si peak broadening.

### Production and characterization of PEO coatings

The samples of AlSi10Mg were degreased in acetone using ultrasounds and then dried with compressed air before the PEO treatment. PEO process was performed in Direct Current (DC) mode with a TDK-Lambda (TDK-Lambda, France) 350 V/8 A power supply, using a carbon steel mesh as the cathode. An aqueous alkaline solution with 25 g/l of Na_2_SiO_3_ and 2.5 g/l of NaOH was employed as electrolyte (Chemicals from Sigma Aldrich, USA). The treatments were performed in galvanostatic mode at 0.5 A/cm^2^ for 10 min. The temperature of the bath was maintained at room temperature through a thermostatic bath. The choice of the PEO process parameters was performed starting from the ones optimized by the authors in previous works^22^ since the aim of this work was not the investigation about the influence of the PEO process parameters, but the identification of the microstructural features of the substrate (and in particular silicon distribution and presence of intermetallic) that affect the characteristics and the properties of coatings. Hence identified the mechanism, this can be reasonably extended to other PEO process parameters. The samples were washed with distilled water and ethanol and dried with compressed air after the PEO treatment. In order to analyze the cross-section, the samples were cut with SiC disk, mounted in epoxy resin, ground with abrasive papers until 4000 grit, and polished with clothes and diamond suspension (6 µm and 1 µm) (all material for metallographic preparation from Cloeren Technology S.r.l., Italy). Cross-sections and surfaces of the coated samples were analyzed by SEM–EDS to evaluate the morphological features, the homogeneity, the composition, and the thickness of the coating. EDS elemental maps were also performed along the cross-section to analyze the elemental distribution into the coating. The phase composition of the PEO layers was evaluated by X-ray diffraction (XRD), using Bruker D8 X-ray diffractometer with a Ni-filtered Cu radiation source, operating at 40 kV and 40 mA, with scans from 15° to 85°, a 0.025 step size and a 5 s dwell time. The measurements were performed in Thin Film mode with a grazing angle of 3°. The microstructural characterization was performed on different PEO coated samples (three for each type of substrate) in order to assure reproducibility of the results.

The mechanical properties of the coatings were evaluated by Vickers micro-hardness tests, performed on polished cross section. The microhardness evaluation was carried out with a Vickers Leitz Wetzlar micro-hardness tester ((Leica Microsystem S.r.l., Milan, Italy), using a load of 100 g. For each sample 10 measures were taken to assure reproducibility.

EIS tests were performed to quantitatively evaluate the corrosion performance of the samples. A Materials Instrument Spectrometer coupled with an AMEL 2549 Potentiostat (Amel S.r.l., Milan, Italy) was used for the EIS analysis that were carried out at the open circuit potential in a frequency range between10^5^ and 10^–2^ Hz, with a perturbation amplitude of 10 mV. Tests were performed using a saturated calomel electrode as reference (SCE) and a platinum electrode as counter in a 0.1 M Na_2_SO_4_ and 0.05 M NaCl solution (chemicals from Sigma Aldrich, USA), to simulate a moderate aggressive environment containing both sulphates and chlorides. The experimental data were fitted with the software Z-view. Moreover, before the test the samples were immersed for 30 min for Open Circuit Potential (OCP) stabilization and the measures were repeated three times to ensure reproducibility. The measures were performed directly after 0 h of immersion and after 24 h of immersion in the same solution used for the EIS test in order to obtain information on the durability of the coatings with the time.

## Results and discussion

In “Analysis of the substrates” section, the results regarding the characterization of the substrates after different heat treatments will be presented. In detail, the samples will be characterized with optical and electron microscopy to study the different silicon distribution induced by the different heat treatments. In “Analysis of PEO coatings” section, the results of the characterization of the PEO coatings will be shown: the microstructural one, performed with SEM and XRD analysis, the mechanical and corrosion one performed with microhardness and EIS test. The microstructure of the obtained coating will be linked with the microstructure of the substrates, presented in “Analysis of the substrates” section, whereas the mechanical and corrosion performance of the coating will be related to the PEO microstructure.

### Analysis of the substrates

**Figure 1:**
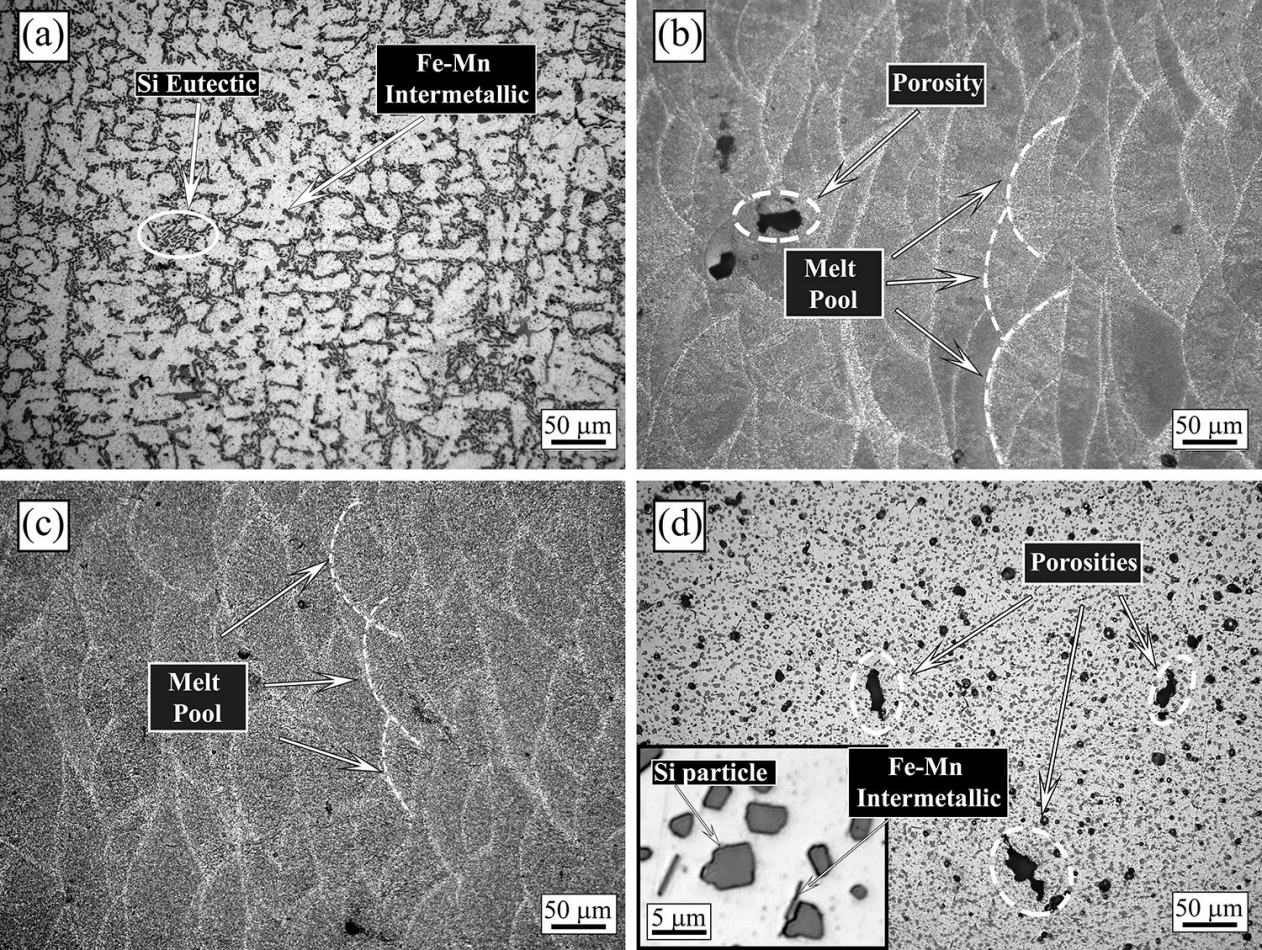
Optical microscope (OM) micrographs of the cast sample (**a**), as printed sample (**b**), annealed sample (**c**), solution treated sample (**d**) obtained at 200 × with Graff-Sargent etching solution.

The L-PBF AlSi10Mg samples, were characterized by OM observations in the as printed condition and after the heat treatments, and compared with the cast AlSi10Mg one (Fig. 1). The microstructure of the cast sample (Fig. 1a) showed the typical dendritic microstructure (the alfa-Al phase with dendritic form is the white one in the micrograph), with the presence of the Si eutectic (highlighted by the circle in the image), and of iron-based intermetallic, as can be usually found for this alloy^10^. In all the L-PBF samples (Fig. 1b–d) some porosities due to the printing parameters were observed. In the sample in as printed condition (Fig. 1b), the typical half-cylindrical melt pools composed of columnar alfa-Al grains surrounded by fine Si particles was observed. This microstructure is the typical one reported in the literature for samples obtained by L-PBF^10^, with the melt pools corresponding to the locally melted and rapidly solidified regions formed during the process.

**Figure 2:**
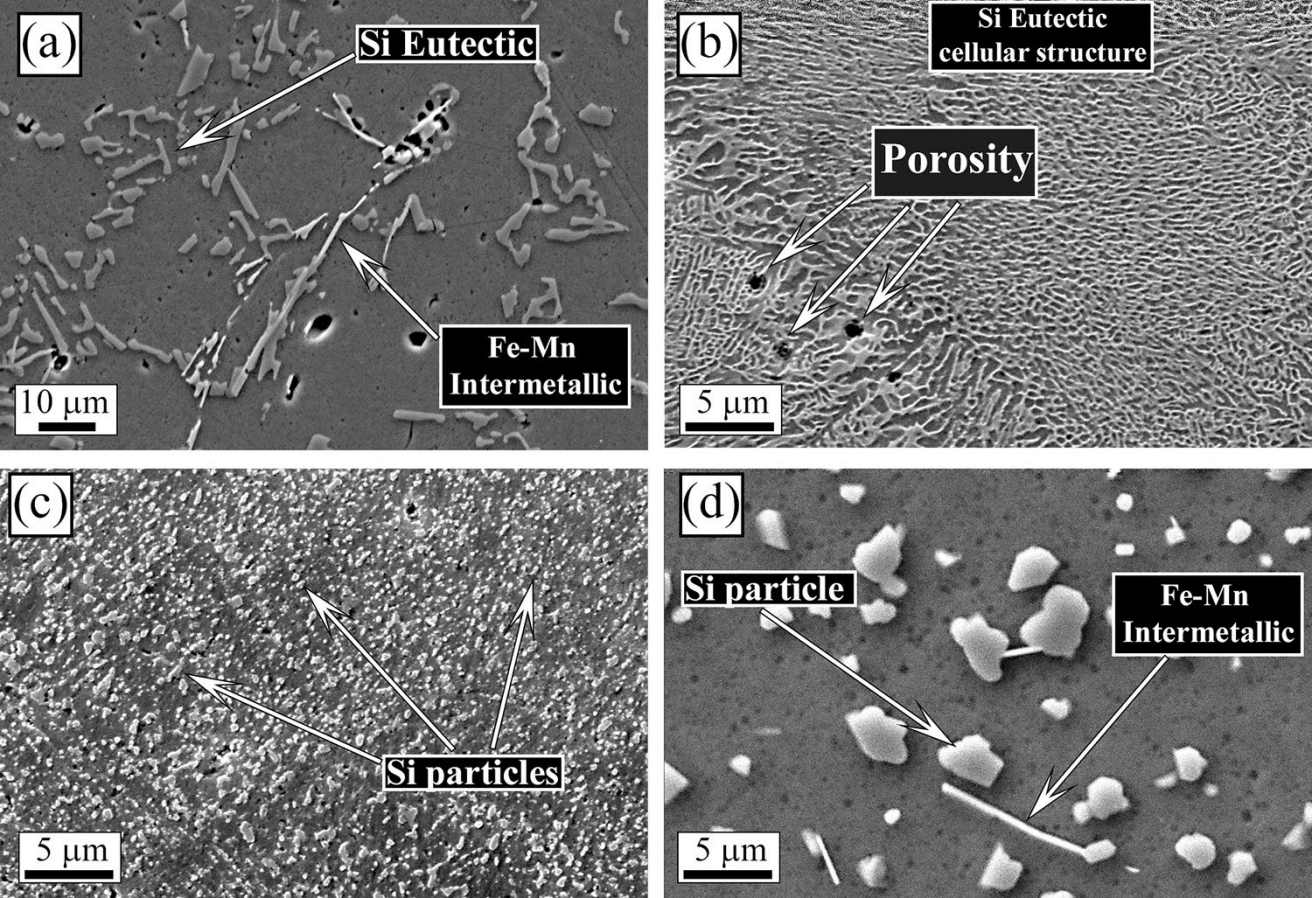
Scanning electron microscope (SEM) images, secondary electron mode, of the cast sample (**a**), as printed sample (**b**), annealed sample (**c**), solution treated sample (**d**) after etching with Graff-Sargent etching solution.

**Figure 3:**
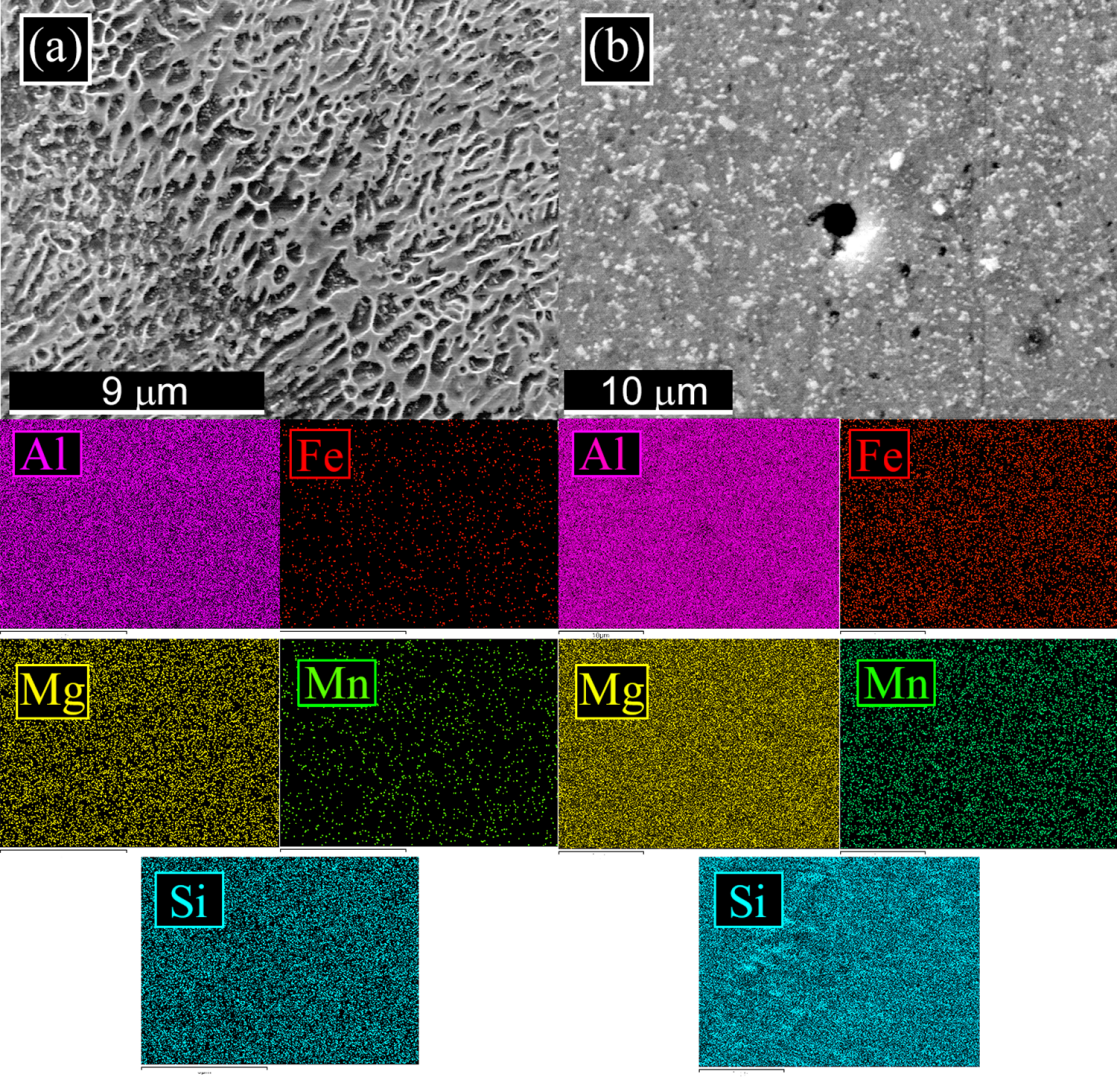
Scanning electron microscope (SEM) images with EDS elemental maps of the as printed sample (**a**) and of the annealed sample (**b**) to highlight the distribution of the elements on the surface.

**Figure 4:**
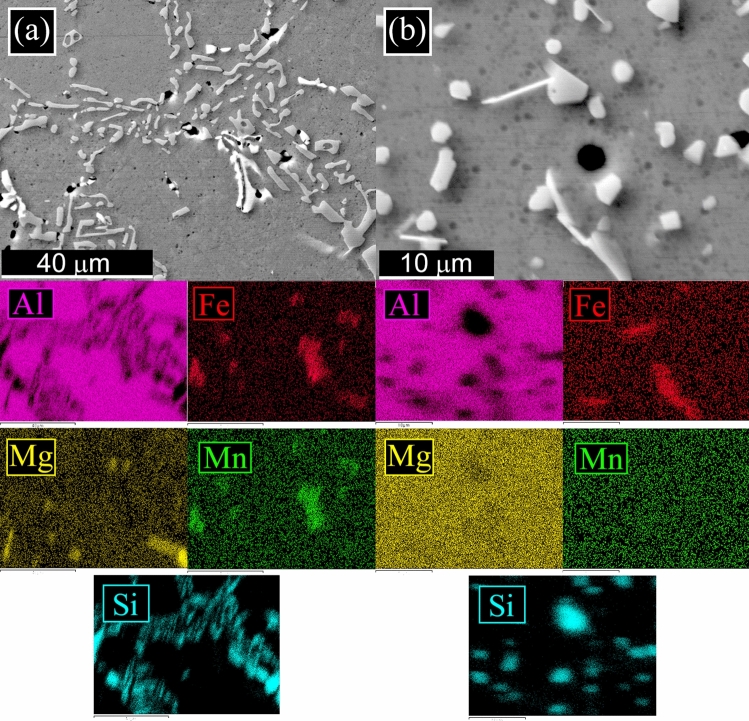
Scanning electron microscope (SEM) images with EDS elemental maps of the cast sample (**a**) and of the solution treated sample (**b**) to highlight the distribution of the elements on the surface.

The L-PBF annealed sample (Fig. 1c) showed the same microstructure of the as printed one, with only a slight coarsening of the Si particles. In particular, according to Takata et al*.*^8^ the fine Si phase precipitates within the columnar α-Al phase and the eutectic Si particles became coarser. Within the α-Al matrix, the developed substructure disappeared, presumably due to the elimination of dislocations occurred during the recovery process at elevated temperatures. Instead, as expected, a completely different microstructure was observed only after solution treatment (Fig. 1d). The melt pools were no longer present and, as shown by the image at higher magnification (Fig. 1d inset), a remarkable coarsening of Si and the formation of Fe-based intermetallic occurred, due to the higher temperature of treatment (530 °C) and longer time of exposure (6 h) that permits to reach thermodynamic equilibrium. Fe–Mn intermetallic were instead not present in the as printed condition, as the extremely rapid solidification process inhibited their precipitation, as well as in the annealed samples where the lower temperature (300 °C) and lower time (2 h) did not permit to reach the equilibrium. SEM analysis of the samples were performed and the results are reported in Fig. 2. In the cast sample (Fig. 2a) was confirmed, by EDS analysis, the presence of the Si eutectic and the Fe–Mn intermetallic, as indicated by the arrows. The L-PBF sample in as printed condition showed the typical cellular structure of L-PBF samples, consisting of fine Si particles (Fig. 2b). From the observation of the annealed sample (Fig. 2c) the cellular structure decomposed by the coarsening of the Si particles. Further coarsening of Si was noted in the solution-treated sample (Fig. 2d) with silicon particles between 1 and 3 µm. In the solution treated sample, the presence of stick-like Fe-based intermetallic sample was confirmed by EDS analysis. In order to better study the silicon distribution into the different samples also EDS elemental maps were performed and the results are reported in Fig. 3 for the as printed (Fig. 3a) and annealed (Fig. 3b) samples and in Fig. 4 for the cast (Fig. 4a) and solution treated (Fig. 4b) samples. The as printed and the annealed samples were characterized by a uniform silicon distribution, with only small zones in the annealed sample in which silicon was more concentrated. These results agreed with SEM analysis that showed a typical cellular structure in the as printed sample and the presence of uniform distributed small silicon particles in the annealed sample. Moreover, the absence of Fe–Mn precipitates was confirmed in both the samples. In the cast and in the solution treated samples (Fig. 4) a completely different elemental distribution was observed. Silicon resulted in fact concentrated in the eutectic zones in the cast sample (Fig. 4a) and in the coarse silicon particles in the solution treated sample (Fig. 4b). In both the samples, the presence of the Fe–Mn precipitates was clearly noted. So, it can be concluded that the as printed and the annealed samples were characterized by a fine uniform silicon distribution and by the absence of Fe–Mn precipitates, whereas in the cast and solution treated samples a coarse non-uniform silicon distribution was detected as also the presence of Fe–Mn precipitates.

**Figure 5:**
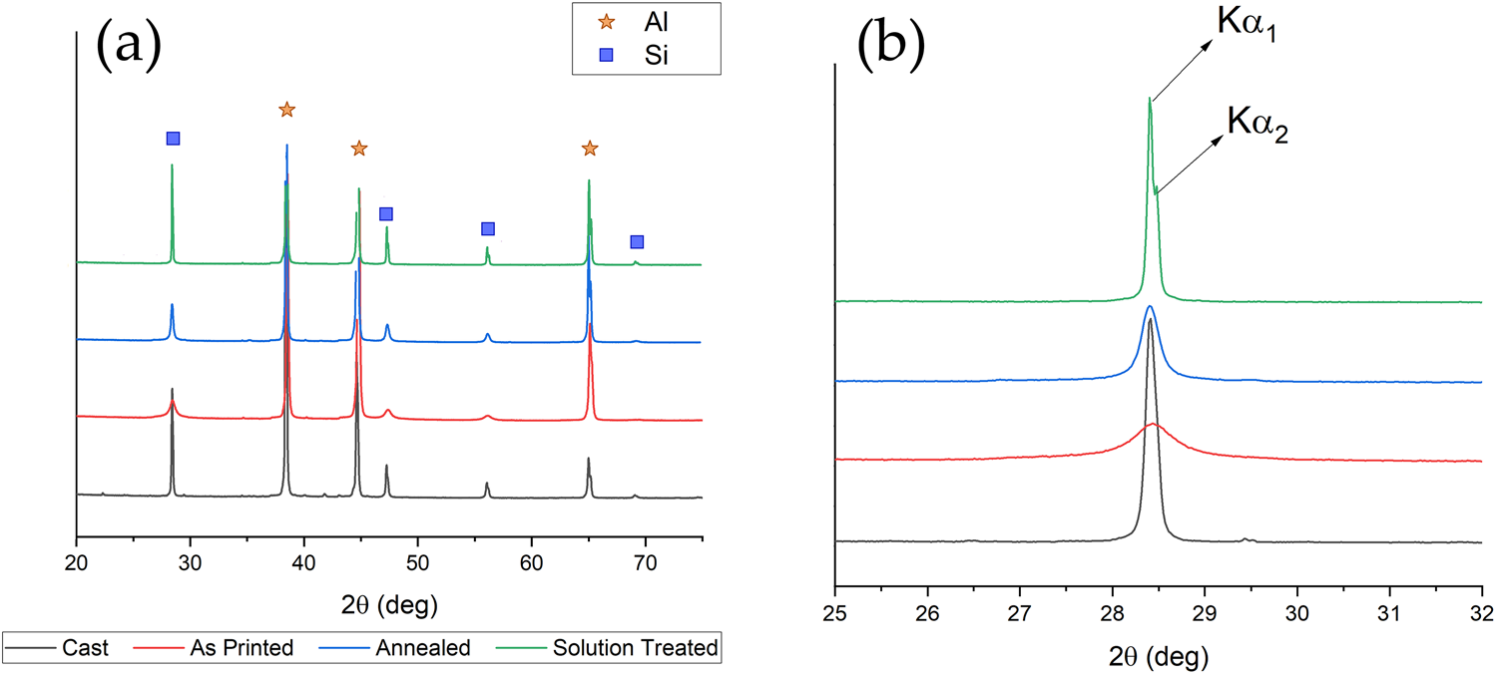
X-ray diffraction (XRD) patterns of the different samples. In (**a**) is reported the peak identification whereas in (**b**) is reported a detail of the first peak of silicon. It can be observed the broadening effect due to the different sizes of the silicon crystallites.

X-ray diffraction analysis were performed on the different samples in order to identify the phases and the results are reported in Fig. 5a. From the reported patterns, the main phases present in the samples were Al and Si. Since from OM and SEM observations, the main difference among the samples was the different dimensions of Si particles, the dimensions of Si crystallites were investigated, by analysis the main peak of Si, located at 28.4°, and the results are reported in Fig. 5b. It can be noted that the XRD results are in accordance with the SEM observations. In fact, the wider peak, and so the smaller size of Si crystallites, was found for the as printed sample, characterized by a very fine Si network that constituted the cellular structure of L-PBF samples. Also the annealed sample, where the Si network resulted destroyed but very fine Si particles were observed, was characterized by a Si wide peak. Instead in the cast and in the solution treated samples, characterized respectively by the presence of the Si eutectic and of large Si particles, the peaks of Si were well defined and not broadened. The obtained results are also in accordance with the ones obtained by Maamoun et al.^43^ after different heat treatments on AlSi10Mg alloy produced by L-PBF, using recycled powders, that showed a decrease in the FWHM of Si peaks with the increase in the size of the Si particles. Also in this work FWHM was calculated and the results are reported in Table 1.

**Table 1:** Average Si FWHM values calculated with the High Score Plus software for the different samples.

	Average Si FWHM (°)
Cast	0.156 ± 0.054
As printed	0.842 ± 0.376
Annealed	0.315 ± 0.132
Solution treated	0.104 ± 0.037

**Figure 6:**
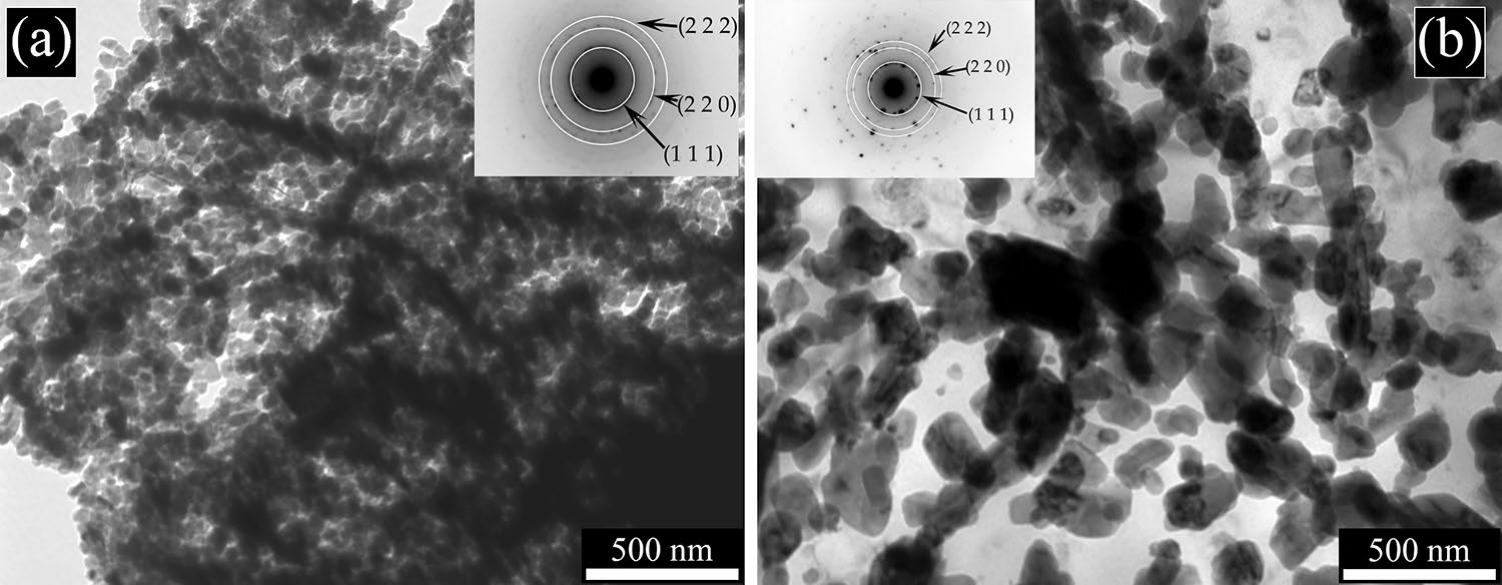
Transmission electron microscope (TEM) images of the as printed sample (**a**) and annealed sample (**b**). Selected area electron diffraction (SAED) pattern is reported in the upper part of the two images.

**Figure 7:**
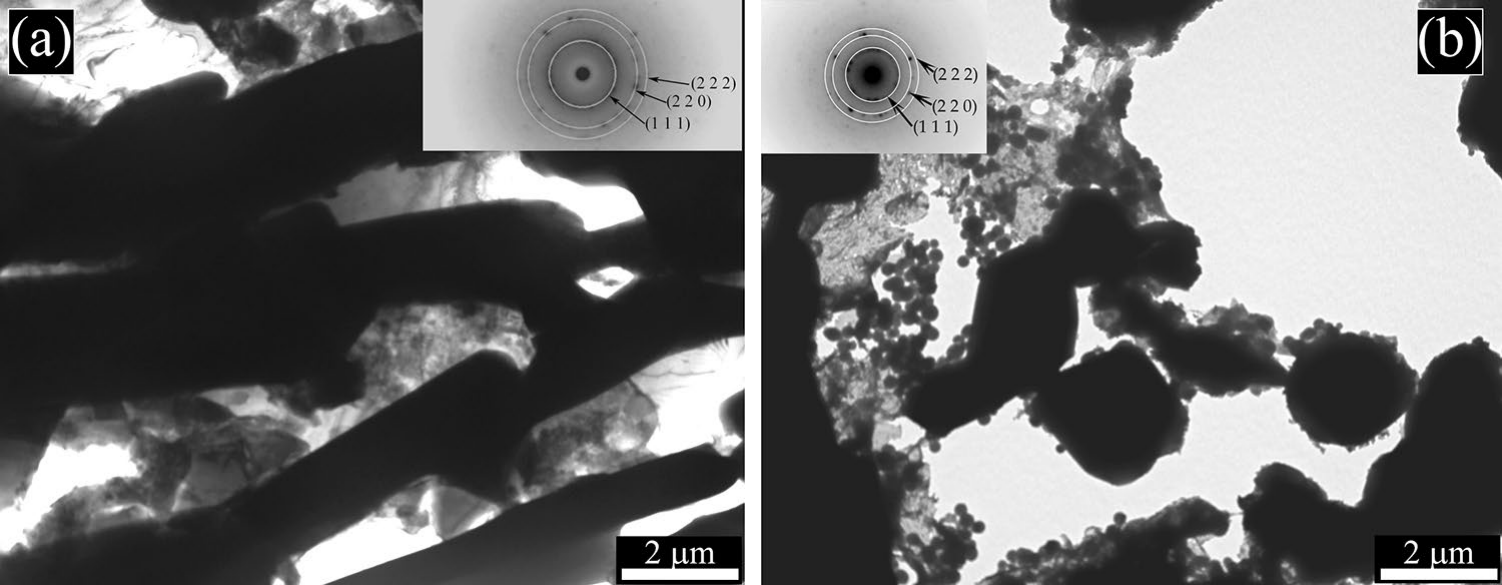
Transmission electron microscope (TEM) images of the cast sample (**a**) and solution treated sample (**b**). Selected area electron diffraction (SAED) pattern is reported in the upper part of the two images.

It is well known that Full Width at Half Maximum (FWHM) is affected by the microstructural features of the material like dislocation density, microstrain, stacking fault, crystallite size etc. In this particular case, crystallite size played an important role. As can be seen from the SEM (Fig. 2) and TEM micrographs (Figs. 6–7), the crystallite size of the as printed alloy showed a very finely distributed network of silicon crystals which was also confirmed by the FWHM of the silicon peaks of the diffraction pattern (Table 1). The as cast sample had a blocky and regular shape of silicon crystals which translates in a FWHM significantly lower compared to that of the as printed sample. Silicon crystals have grown during the annealing treatment, in fact they showed a slightly lower FWHM than the as printed sample and the treatment performed at higher temperature (solution treatment) increased even more the size of the silicon crystals, lowering the FWHM to values comparable to that of the cast material.

In order to study the silicon distribution in the different samples, also TEM analysis was performed and the results of as printed and the annealed samples are reported in Fig. 6, and of cast and solution treated samples in Fig. 7. In all the micrographs the black zones are the silicon particles or the silicon network, confirmed by the SAED pattern, reported in the upper part of the images. Remarkable differences in the Si distribution were observed in the various samples. In both samples reported in Fig. 6, a fine distribution of silicon can be noted. In particular, in the as-printed sample (Fig. 6a) can be observed that the cellular network is made of very fine silicon particles (about 50 nm). The size of the Si particles increases with the annealing treatment (Fig. 6b) but these remain of sub-micrometric dimensions (around 250 nm). In the solution-treated sample (Fig. 7b) the size of the Si particles dramatically increases, as already demonstrated also by SEM analysis, with an average size of about 2 µm. In the cast sample (Fig. 7a) rod-like Si network, derived from eutectic Si can be observed with dimensions further increased in comparison to the solution treated sample.

### Analysis of PEO coatings

**Figure 8:**
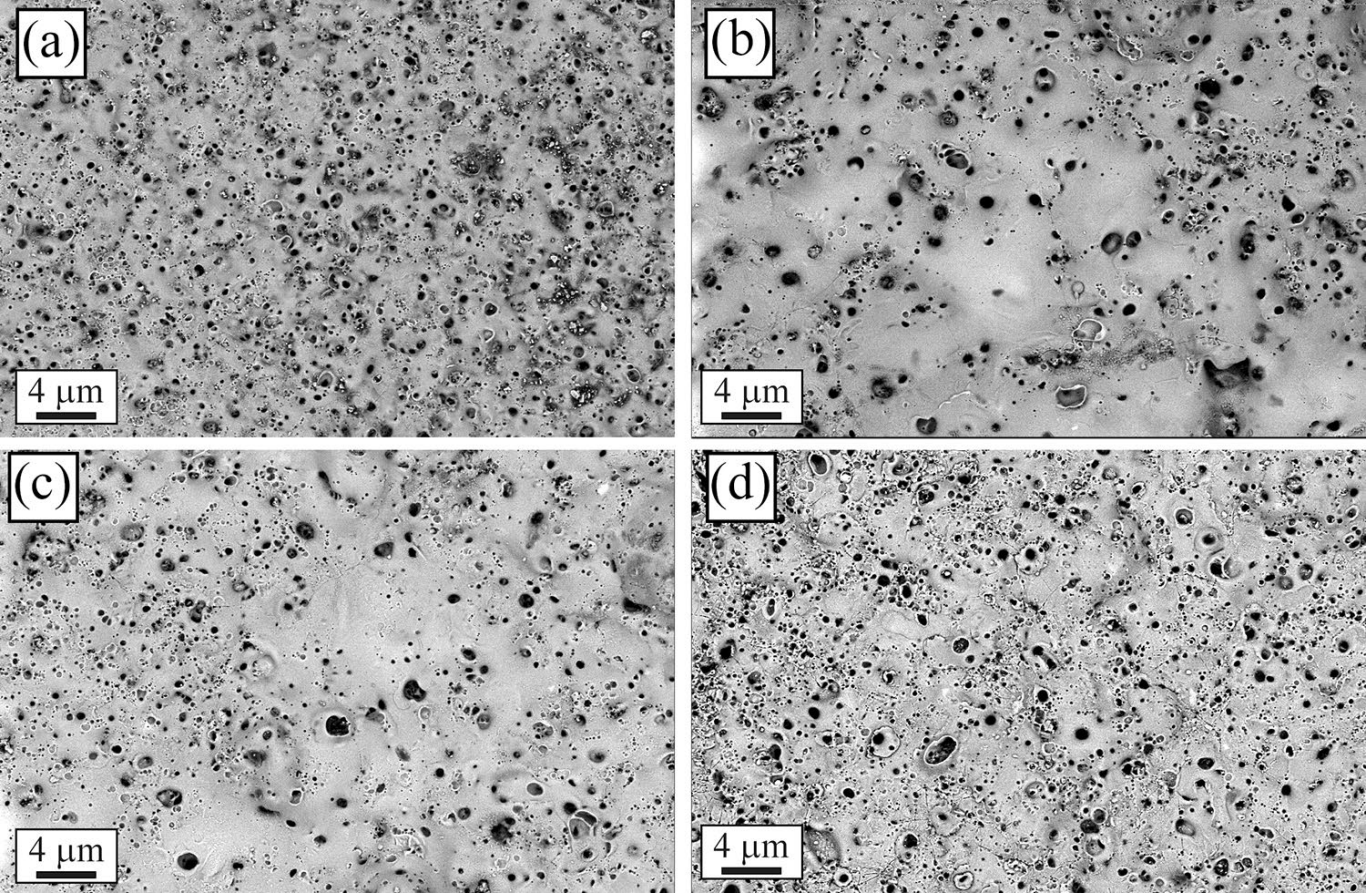
Scanning electron microscope (SEM) images, backscattered electron mode, of the surfaces of the samples after PEO treatment: cast sample (**a**), as printed sample (**b**), annealed sample (**c**), and solution treated sample (**d**).

**Figure 9:**
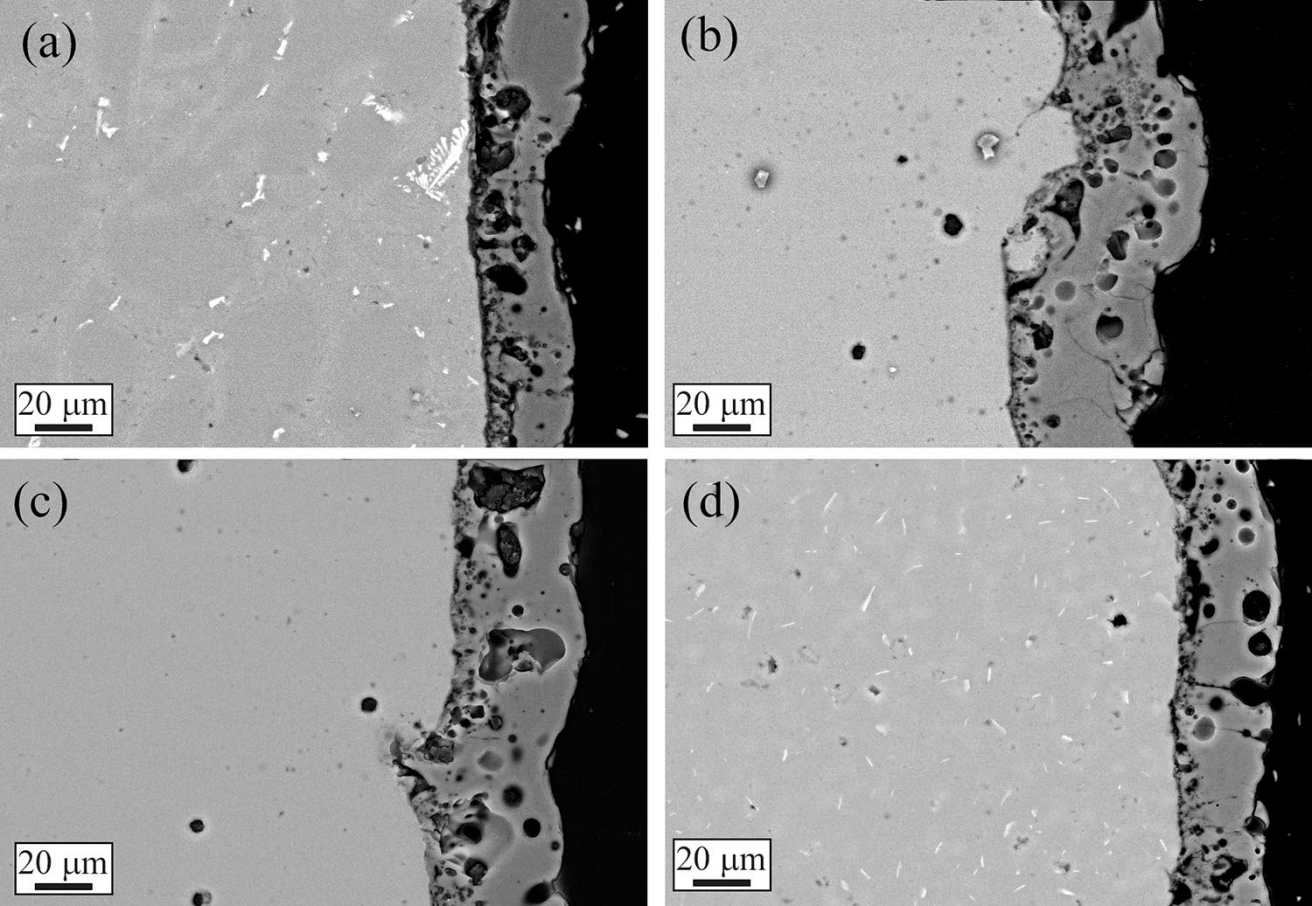
Scanning electron microscope (SEM) images, backscattered electron mode, of the cross-sections of the samples after PEO treatment: cast sample (**a**), as printed sample (**b**), annealed sample (**c**), and solution treated sample (**d**).

PEO coatings were produced using as substrate the as cast AlSi10Mg and the L-PBF-produced AliSi10Mg samples, in the condition as printed and after the annealing and solution treatment, to investigate the effects of the different microstructures on the structure of the obtained PEO layers. The SEM images of the surfaces and the cross-sections of the coatings produced on the different samples are shown in Figs. 8 and 9, respectively. Considering the surfaces of PEO coated samples (Fig. 8), all the samples were characterized by the typical microstructure of PEO coatings with the presence of pancake structures and volcano-like pores that derived from the formation of micro-discharges during PEO treatment^44^. The main difference among the samples was the porosity that was higher in the cast (Fig. 8a) and solution treated (Fig. 8d) samples, in comparison to the as printed (Fig. 8b) and annealed (Fig. 8c) samples. This fact is in accordance with a previous work of the authors^38^, where it was found that Fe-rich intermetallic, in this work present only in the as-cast and solution treated samples, along with large Si eutectic induced the formation of a higher porosity in the coating due to the different electrochemical behavior of the phases. Considering the cross-section of the samples (Fig. 9) also in this case the main differences can be found between the cast and solution treated samples (Fig. 9a, d respectively) and the as printed and annealed samples (Fig. 9b and c respectively). In fact, the cast and solution treated samples were characterized by a coating about 25 µm thick, with the presence of defects and porosities at the interface between the substrate and the coating, whereas the as-printed and annealed sample showed a thicker coating (about 35 µm) that resulted also more uniform at the interface with the substrate. This behavior can be also in this case related to the microstructure of the substrate: in the cast and solution-treated samples were present Fe-rich intermetallic and large Si particles, all characterized by different electrochemical behavior compared to the Al matrix, thus affecting the kinetic of the oxide growth. This fact resulted also in accordance with Li et al.^41^ who found that a refinement in the Si phase in the substrate produced an increase in the thickness of PEO layers. The presence of defects at the interface between the metal and the coating in the cast and solution treated samples, was related to the presence of the Fe-rich intermetallic in these samples. A matter of fact, in Fig. 9a where a remarkable defect was observed in the correspondence of the white Fe-rich intermetallic. The as-printed and annealed samples were instead characterized by a fine Si cellular structure and by fine Si particles, that allowed a better distribution of the micro-discharge during the PEO process, and, therefore, the formation of a thicker coating with a reduced number of defects at the interface substrate-coating. Therefore, the different microstructures of the coatings were related to the silicon distribution and to the presence of Fe–Mn precipitates, due to the different electrochemical behavior of large Si particles, compared to the fine and homogeneously distributed Si, and of the precipitates. Regarding the influence of Si distribution, accordingly also to Li et al.^41^, the oxide protective layers in Al-Si started to form on the aluminum matrix. If the substrate is characterized by coarse silicon particles, as in the case of the cast and solution treated samples (see TEM analysis in Fig. 7), the formed oxide layer on Al substrate does not cover the adjacent large Si phase, thus preventing the growth of a uniform layer on the surface of Al-Si alloy. If instead Si is uniformly distributed at sub-micrometric scale, as in the as printed and the annealed samples (as confirmed by the TEM analysis of Fig. 6), the surrounding Al matrix is quickly oxidized and the formed oxide layer fully covers most of nearby small Si particles. Considering Fe–Mn precipitates, according to Wang et al.^42^, the presence of Fe produces residual defects in the native oxide layer providing a path for the penetration of electrolyte, and thus causing the formation of more porous coatings and reducing the growth rate of the coating, due to a reduction in the oxidation efficiency. For these reasons, the coatings obtained on the as printed and annealed samples, characterized by a uniform and finely distributed silicon and by the absence of Fe–Mn precipitates, resulted thicker and denser. Considering that the samples obtained by L-PBF are always characterized by a fine cellular structure of the eutectic silicon and by the absence of Fe–Mn precipitates, these results can to explain the different microstructure of the coating obtained on the L-PBF samples in comparison with the cast samples and can be extended also to other PEO process parameters.

Usually PEO coatings are constituted by two different sub-layers: a denser barrier layer, near the substrate, that give the main protection against corrosion, and an external porous or technological layer, that can be employed to proper functionalize the surface thank to presence of the pores^33,34^. In this case differences in the relative thickness of the two sub-layers can be identified in the various samples. In particular the thickness of the non-porous sublayer of the printed samples (as printed, annealed and solution treated) is less (up to about 2.5 µm) than that of the cast sample (up to about 3.3 µm), as can be observed in Fig. 9. This can be probably linked to the fact that in the printed samples a large amount of silicon is included in the reaction region of the micro discharge due to the printing process. As a result, the oxidation of aluminum decreases and the density of the coating decreases with the barrier layer becoming thinner.

**Figure 10:**
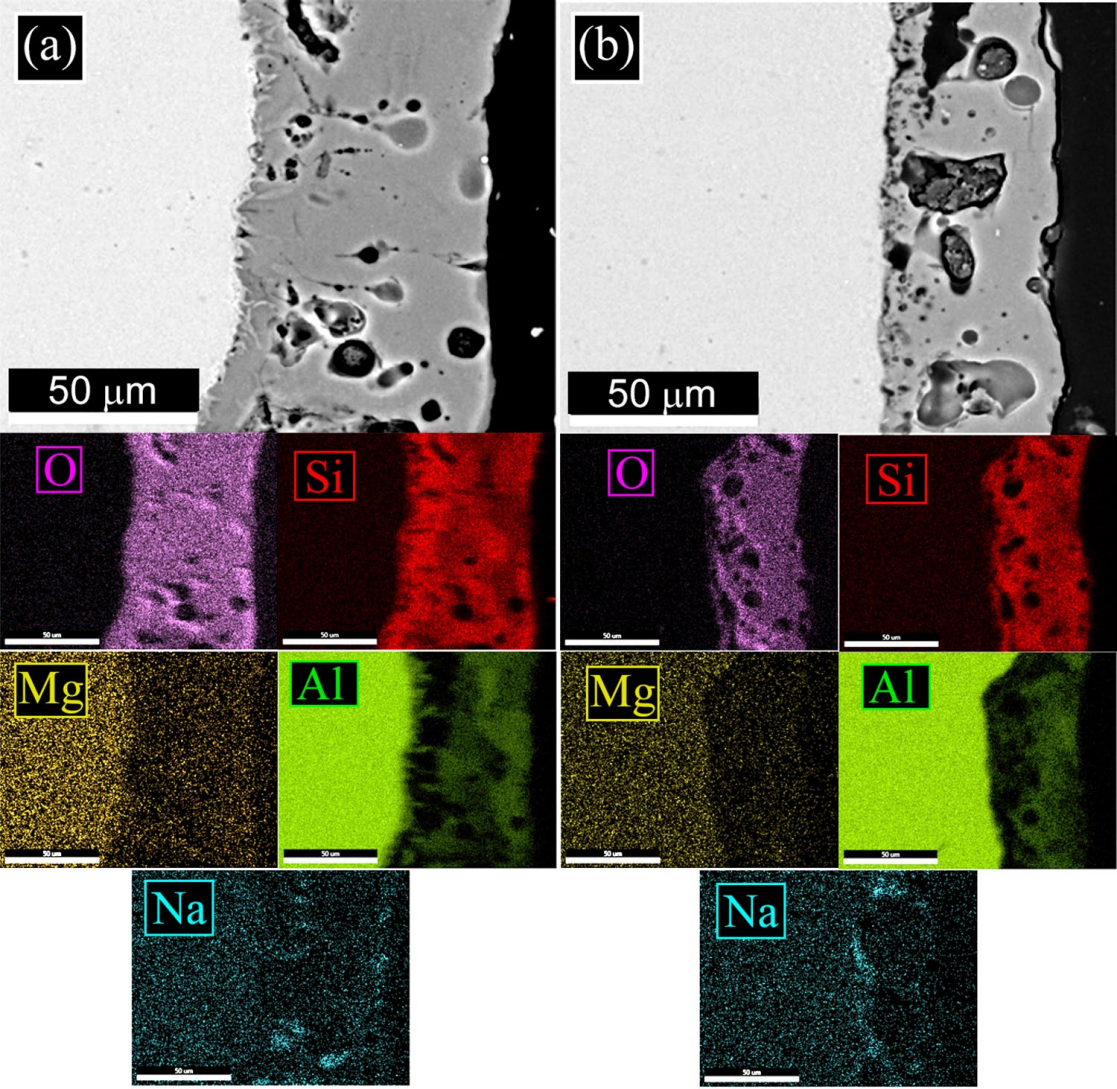
SEM–EDS elemental mapping of the cross-sections of the samples after PEO treatment: as printed sample (**a**) and annealed sample (**b**).

**Figure 11:**
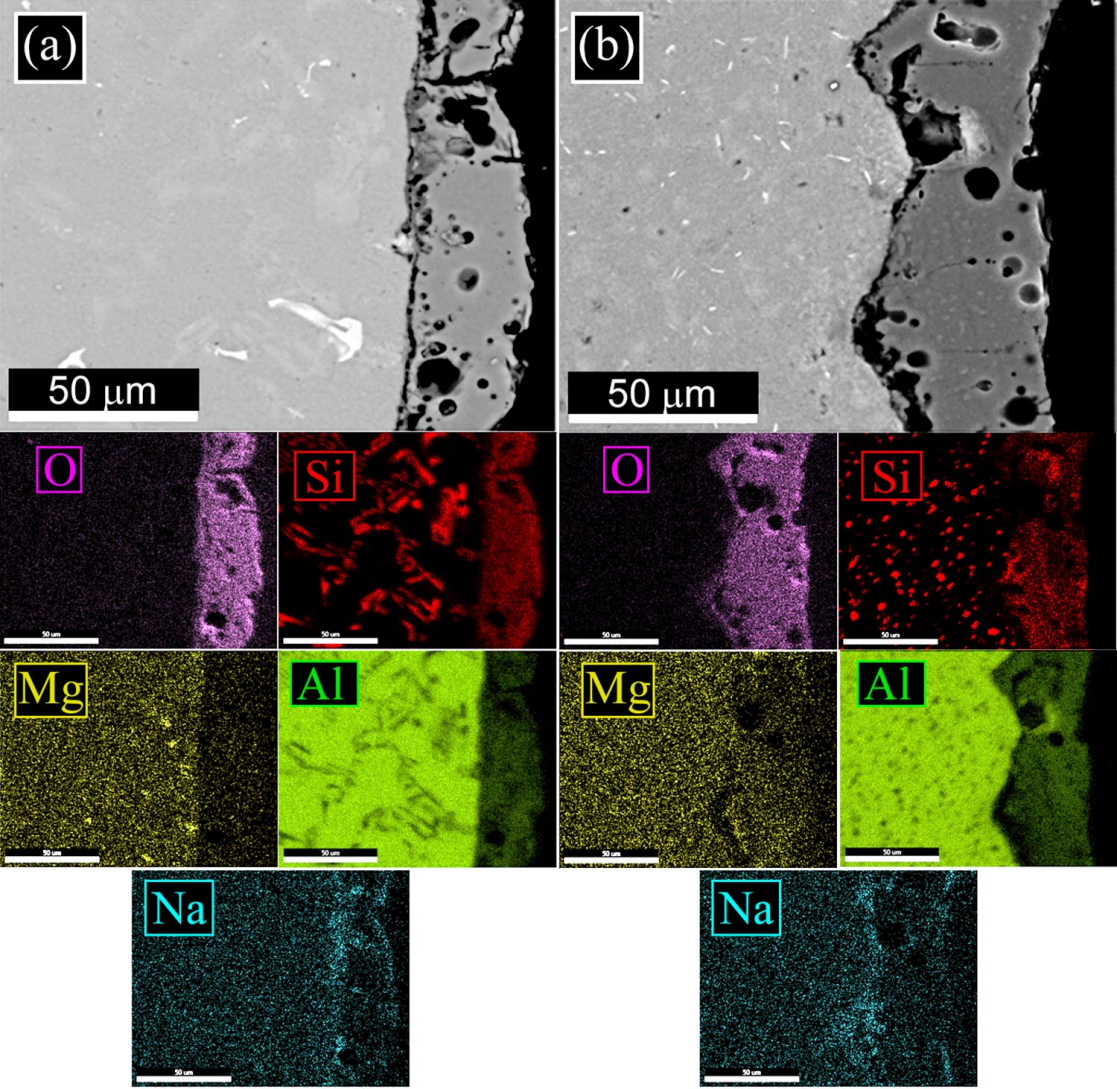
SEM–EDS elemental mapping of the cross-sections of the samples after PEO treatment: cast sample (**a**) and solution treated sample (**b**).

To better study the influence of the microstructure of the substrate on the PEO layers, also EDS elemental mapping was performed on the cross-sections of the coated samples. In detail, in Fig. 10 can be found the maps for the as-printed and the annealed samples, whereas in Fig. 11 are reported the maps for the cast and solution treated samples. Generally, from the literature, the homogeneity in the distribution of the elements in the PEO coatings is related to the microstructure of the substrate and to the electrical parameters employed during the treatment. In detail, an increase in the silicon content in the coating was observed near the zones of the substrate enriched in silicon^40^. Moreover, an increase in the duty cycle or the use of direct current produced an increase in the inhomogeneity of silicon into the coating^45^. In the present work, the Si elemental distribution in the PEO coatings of the as printed and annealed samples (Fig. 10a, b) resulted homogeneous. This was related to the homogeneous microstructure of the substrate with the presence of the typical cellular network of silicon in the as-printed sample and finely dispersed particles of silicon in the annealed samples, as evidenced previously in Fig. 3. Different situations can be observed in the cast sample (Fig. 11a) and the solution-treated sample (Fig. 11b). In these samples, the silicon distribution in the substrate was very inhomogeneous with the presence of the eutectic in the cast sample and of large silicon particles in the solution-treated sample, as was observed in Fig. 4. This produced a quite inhomogeneous distribution of silicon in the PEO layers: Silicon in the coating was more concentrated near the eutectic or the Si large particles of the substrate. This behavior resulted also in accordance with the ones found from the authors in a previous work^38^.

In order to deeply investigate the differences in EDS maps of PEO coatings, with the different substrates, also semi-quantitative EDS analysis were performed on the surfaces and cross sections of the PEO treated samples, and the results are reported in Table 2. The composition of the surfaces resulted similar in the various samples, except for the sodium content that resulted higher in the as cast and in the solution treated samples. It can be also observed, in accordance with what observed from EDS maps in Figs. 10 and 11, a decrease in the silicon content in the cross section of the as cast and the solution treated samples in comparison with the as printed and the annealed sample.

**Table 2:** Semi-quantitative EDS analysis performed on the surfaces and the cross sections of the different PEO treated samples (values in wt%).

	O%	Al%	Si%	Na%
Cast surface	45.6	11.3	34.9	8.2
Cast cross section	41.9	17.3	38.3	2.5
As printed surface	50.4	15.0	32.2	2.4
As printed cross section	45.6	6.6	43.8	4.0
Solution treated surface	48.3	11.9	32.5	7.3
Solution treated cross section	42.1	18.0	38.1	1.8
Annealed surface	49.2	13.1	33.1	4.6
Annealed cross section	45.7	11.5	40.3	2.4

**Figure 12:**
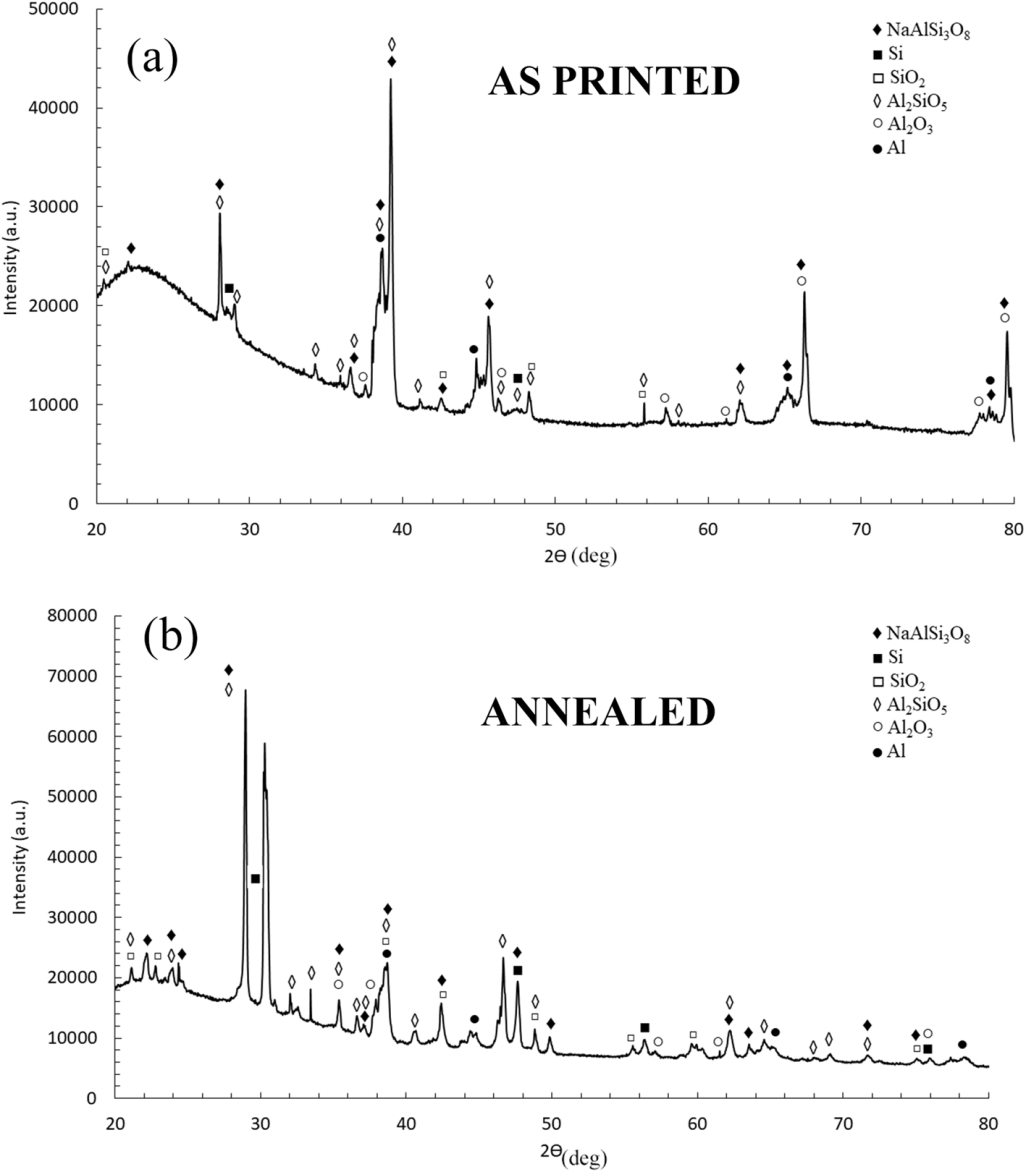
X-ray diffraction (XRD) pattern of PEO treated samples: As printed sample (**a**); annealed sample (**b**).

**Figure 13:**
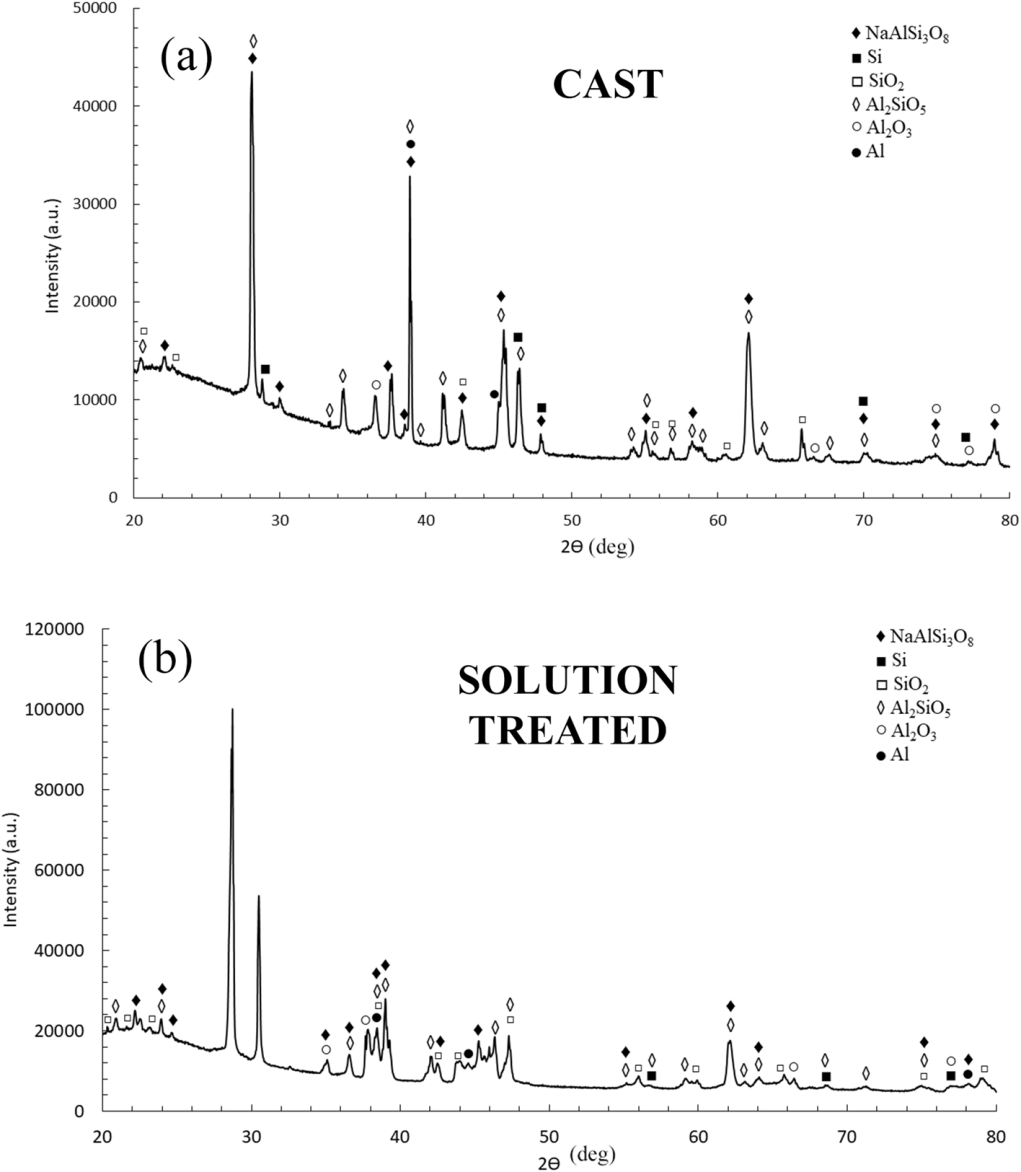
X-ray diffraction (XRD) pattern of PEO treated samples: cast sample (**a**); solution treated sample (**b**).

In order to clarify if the different microstructure influenced also the phase composition of the coatings X-ray diffraction analysis was performed on the different PEO coated samples and the results are reported in Figs. 12 and 13. In all the samples, the peaks of Al and Si, coming from the reflection from the substrate, can be observed. Moreover, the presence of SiO_2_, Al_2_O_3_, Al_2_SiO_5_ (kyanite), and NaAlSi_3_O_8_ was observed, in agreement with the literature^46,47^. The more important difference among the patterns was the quantity of amorphous phase that was remarkably higher in the as-printed sample (Fig. 12a), in comparison to the others. In fact, whereas the pattern of the cast, annealed and solution treated samples were similar, in the as-printed one an evident amorphous phase at low angles with less pronounced peaks resulted. A higher component of the amorphous phase in PEO coatings formed on AM samples, in comparison with the ones obtained on cast samples, was already found by the authors^22^ and Rogov et al.^36^ and it, generally, was related to the more homogeneous microstructure of the 3D printed alloy. The TEM results of this work showed that in the as printed samples was present a nano-cellular network of silicon, homogenously dispersed in the matrix of aluminum. This structure, promoting a simultaneous oxidation of Al and Si, can be considered the responsible of the formation of a higher amorphous fraction. As matter of fact, in the other type of samples, characterized by the presence of more isolated and larger silicon particles, the amorphous fraction is less pronounced.

In order to investigate whether the microstructural differences influence the mechanical properties of the coatings, Vickers micro-hardness tests were performed along the cross section on both the substrate and the coating (Table 3). Considering the substrates, the hardness of the cast and heat treated were samples characterized by lower hardness in comparison with the as printed one, in agreement with literature^8^. Considering the coatings, the higher hardness was measured in the coating of printed sample (882 HV), followed by the one of annealed sample (788 HV) and by ones of the cast and solution treated samples (524 and 551 HV, respectively). These results can be related to different factors. First of all, the coating produced on the as printed sample was the one characterized by the higher amorphous phase, as evidenced by XRD in Fig. 12, and this induces an increase in the mechanical properties, as evidenced by Pillai et al.^48^. Secondly, as evidenced by the SEM observations of both the surfaces and the cross sections, the coatings produced on the as printed and the annealed samples were thicker and denser, thus leading to higher hardness values in comparison to the ones obtained on the cast and the solution treated samples.

**Table 3:** Vickers micro-hardness (100 g load) obtained on the substrate and on the cross section of the PEO coatings in the different samples.

	HV_0.1_
Cast substrate	27.5 ± 1.3
Cast PEO coating	524.1 ± 24.8
As printed substrate	128.1 ± 6.2
As printed PEO coating	882.4 ± 25.1
Solution treated substrate	66.2 ± 4.8
Solution treated PEO coating	551.4 ± 20.3
Annealed substrate	68.0 ± 4.5
Annealed PEO coating	788.0 ± 24.6

**Figure 14:**
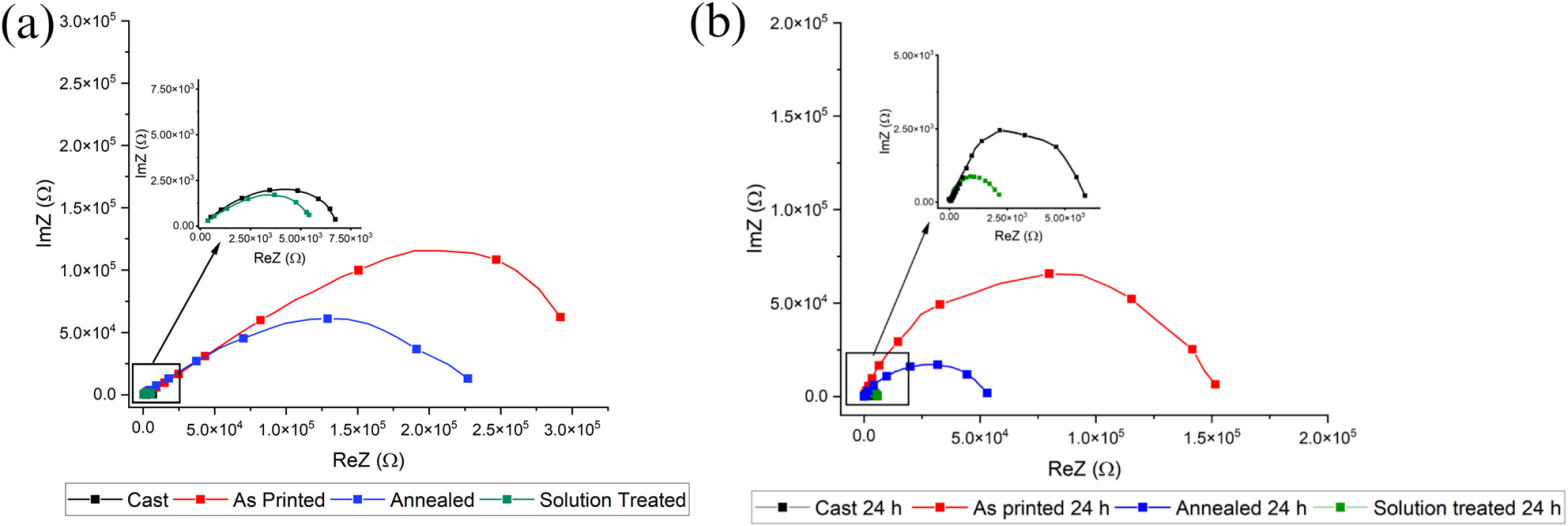
Results of the EIS tests, in term of Nyquist plots, for the different PEO coated samples after 0 h of immersion (**a**) and after 24 h of immersion (**b**). At the high left a zoom of the zone of the graph highlighted by black box. Test Electrolyte: 0.1 M Na_2_SO_4_ and 0.05 M NaCl.

**Figure 15:**
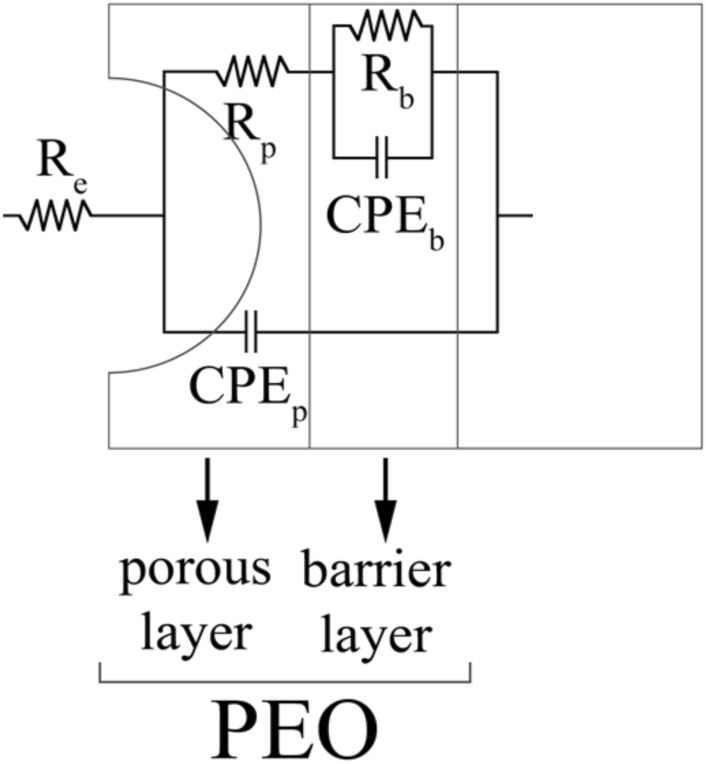
Equivalent circuit used to fit the coming from EIS tests.

In order to quantitatively evaluate the corrosion performance of the samples and to evaluate whether the recorded differences in the microstructure of the coatings influence the corrosion resistance of the coatings, EIS tests were performed in a moderate aggressive electrolyte. The results in term of Nyquist plot are reported in Fig. 14, where dots represent the experimental data. In detail in Fig. 14a are presented the results of the EIS tests after 0 h of immersion whereas in Fig. 14b the ones after 24 h of immersion in the same electrolyte used for the tests. The recorded data were also fitted using the circuit reported in Fig. 15 and the results of the fitting, that are graphically represented in Fig. 14 as dashed lines, can be found in Table 4 for the samples after 0 h of immersion and in Table 5 for the samples after 24 h of immersion. The choice of the equivalent circuit was performed on the basis of the literature on PEO coatings^49^ that suggests to employ a double circuit (Fig. 15) to fit data coming from PEO treated samples in order to consider the presence of an inner and an external layer. Considering the physical meaning of the different elements of the equivalent circuits in Fig. 15, R_e_ represents the resistance of the electrolyte, R_p_ and CPE_p_ represents the porous layer of PEO coating whereas R_b_ and CPE_b_ the barrier layer. Constant phase elements (CPEi) were used in the equivalent circuits instead of capacitances due to the fact that the measured capacitance is not ideal. In the Nyquist plot the real part of the impedance at the low frequencies (interception with the X-axis) can be considered as qualitive measure of the corrosion properties in terms of polarization resistance and from the observation of Fig. 14 can be clearly noted that the as printed and the annealed samples are characterized by a polarization resistance two order of magnitude higher than the one of the solution treated and cast sample (visible in the zoom at the high left). This result is clear both after 0 h of immersion (Fig. 14a) and after 24 h of immersion (Fig. 14b). After 24 h of immersion can be observed for all the samples a reduction in the polarization resistance, due to the penetration of the electrolyte into the pores that characterize the PEO layer. Considering the results of the fitting of the experimental data for the samples after 0 h of immersion, reported in Table 4, it can be noted that both the values of R_p_ and R_b_ are higher for the as printed and the annealed samples (with R_p_ in the order of 10^4^ and R_b_ in the order of 10^5^) than for the cast and solution treated samples, characterized by values of R_p_ and R_b_ in the order of 10^3^. The corrosion performances can be related with the microstructural observations, in fact in the as printed and annealed samples the PEO layers resulted thicker, denser and with more homogeneous composition, thus producing improved corrosion performances in comparison with the coatings produced on the cast and solution treated samples. Considering the other parameters can be observed that Q_B_ and Q_P_ remain at the same level in all the samples but a significant reduction in n_B_ value can be observed in the as printed and annealed samples. This means that the barrier layer density has increased, but the capacitance Q_B_ is the same, hence the barrier layer thickness has decreased in the as printed and annealed samples, as evidenced also by the SEM observation previously reported. The electrochemical tests so confirm the SEM observations.

**Table 4:** Results of the fitting of the experimental data coming from EIS tests on the samples after 0 h of immersion.

Sample	${\mathrm{R}}_{\mathrm{S}}$(Ω * ${\mathrm{cm}}^{2}$)	${\mathrm{R}}_{\mathrm{p}}$(Ω * ${\mathrm{cm}}^{2}$)	${\mathrm{R}}_{\mathrm{b}}$(Ω * ${\mathrm{cm}}^{2}$)	Q_B_ (F cm^−2^_Hz_^1−n^)	${\mathrm{n}}_{\mathrm{B}}$	Q_P_ (F cm^−2^_Hz_^1−n^)	${\mathrm{n}}_{P}$	${\upchi }^{2}$
Annealed	122	14,520	2.3 × ${10}^{5}$	7.22 × ${10}^{-6}$	0.75	5.55 × ${10}^{-6}$	0.89	8.8 × ${10}^{-7}$
As printed	123	24,187	3.5 × ${10}^{5}$	1.91 × ${10}^{-6}$	0.66	3.45 × ${10}^{-6}$	0.73	1.0 × ${10}^{-5}$
Cast	123	3394	7697	2.24 × ${10}^{-7}$	0.83	3.71 × ${10}^{-6}$	0.64	3.9 × ${10}^{-5}$
Solution treated	120	668	5084	1.16 × ${10}^{-7}$	0.98	3.85 × ${10}^{-6}$	0.65	1.7 × ${10}^{-4}$

**Table 5:** Results of the fitting of the experimental data coming from EIS tests on the samples after 24 h of immersion.

Sample	${\mathrm{R}}_{\mathrm{S}}$(Ω * ${\mathrm{cm}}^{2}$)	${\mathrm{R}}_{\mathrm{p}}$(Ω * ${\mathrm{cm}}^{2}$)	${\mathrm{R}}_{\mathrm{b}}$(Ω * ${\mathrm{cm}}^{2}$)	Q_B_ (F cm^−2^_Hz_^1−n^)	${\mathrm{n}}_{\mathrm{B}}$	Q_P_ (F cm^−2^_Hz_^1−n^)	${\mathrm{n}}_{\mathrm{P}}$	${\upchi }^{2}$
Annealed 24 h	118	11,220	5.5 × ${10}^{4}$	4.12 × ${10}^{-6}$	0.78	3.52 × ${10}^{-6}$	0.75	9.1 × ${10}^{-8}$
As printed 24 h	115	19,452	1.6 × ${10}^{5}$	2.81 × ${10}^{-6}$	0.71	4.86 × ${10}^{-6}$	0.78	2.0 × ${10}^{-6}$
Cast 24 h	120	1423	5240	7.21 × ${10}^{-7}$	0.79	6.85 × ${10}^{-6}$	0.69	1.2 × ${10}^{-6}$
Solution treated 24 h	121	442	2510	2.42 × ${10}^{-7}$	0.85	4.38 × ${10}^{-6}$	0.70	2.8 × ${10}^{-5}$

Considering the results of the fitting of the EIS tests performed after 24 h of immersion (Table 5) it can be confirmed a remarkable reduction in the values of polarization resistance of the coatings. In particular both the values of R_B_ and R_P_ resulted at least halved after 24 h of immersion, due to the fact that without any sealing treatment the electrolyte can enter the pores that characterize the PEO layer and consequently reduce the global resistance of the coating. Regarding the other parameters such as Q and n no significant differences can be recorded after 24 h of immersion. Considering this remain valid the considerations regarding the thickness of the barrier and porous layer after 0 h of immersion, with the barrier layer that results thinner in the AM samples even after 24 of immersion. This can also explain the fact that the higher reduction in the values of polarization resistance can be observed in the annealed samples, that were the samples were the barrier layer resulted thinner.

## Conclusions

In the present work, the effect of the microstructure of the substrate on PEO coatings produced on SLM AlSi10Mg alloys was investigated. In particular, were produced PEO coatings on samples obtained by SLM in the as-printed condition and after an annealing or a solution treating treatment. For comparison, PEO coating was also produced on as-cast sample.

The main findings can be summarized as follow:

- The microstructure of the substrate strongly influenced the formation of the PEO layer. In particular, in the samples characterized by the presence of Fe-rich precipitates and large Si particles or Si eutectic (cast sample and solution treated sample), the PEO coating resulted thinner and more porous than in the other samples. This fact was related to the non-uniform distribution of the micro-discharges during the coating formation, due to the different electrochemical behavior among the Si particles, the Fe-rich intermetallic and the Al matrix.
- Also, the elemental distribution into the coating was strongly influenced by the microstructure of the substrate. In particular, the as-cast and solution treated samples, characterized by a non-uniform distribution of silicon in the substrate showed a non-uniform distribution of Si into the PEO layer.
- Also phases distribution resulted influenced by the microstructure of the substrate. The nano-cellular network of silicon in the as-printed sample induced the formation of a coating with a higher amorphous fraction. In all the other samples, regardless the dimension of the silicon particles, a more crystalline coating was formed, suggesting that the increase in the amorphous fraction was related to the particular microstructure in as printed sample, produced by SLM.
- The microstructure of the substrate, and in particular the silicon distribution, strongly influenced the microstructure and the performances of PEO coatings.
- The coating produced on the as printed samples resulted the best in terms of mechanical properties due to the presence of higher quantity of amorphous phase.
- The coatings produced on the as printed an annealed sample resulted the best in terms of corrosion performances, due to their homogeneity, higher thickness and density.
- The unique microstructure of samples produced by L-PBF determined the microstructure and so the properties of the PEO coatings, reasonably independently by the parameters employed for the PEO treatment.

## Data Availability

The datasets generated and/or analysed during the current study are not publicly available due part of an ongoing study but are available from the corresponding author on reasonable request.
